# Genome-wide DNA methylation analysis of breast cancer MCF-7 / Taxol cells with MeDIP-Seq

**DOI:** 10.1371/journal.pone.0241515

**Published:** 2020-12-11

**Authors:** Ying Shi, Weihua Gong, Xiangrong Gong, Ping Wang, Xin Zhao

**Affiliations:** 1 Clinical Laboratory, The Third Affiliated Hospital of Zhengzhou University, Zhengzhou, China; 2 Internal Medicine Department, The First Affiliated Hospital of Zhengzhou University, Zhengzhou, China; 3 Department of Radiology, The Third Affiliated Hospital of Zhengzhou University, Zhengzhou, China; Cleveland Clinic Lerner Research Institute, UNITED STATES

## Abstract

Breast cancer (BC) is the most frequently diagnosed tumor in women worldwide. Although the combination of surgery and Taxol chemotherapy can achieve a certain therapeutic effect, patients often develop drug-resistance, resulting in a poor prognosis. Therefore, it is significative to seek the molecular mechanism of chemotherapy resistance. Recent studies have found that abnormal epigenetic regulation in breast cells changes the expression of key genes, which can lead to the occurrence, development, and maintenance of cancer, even related to the development of drug-resistance. Therefore, in this study, we performed methylated DNA immunoprecipitation-sequencing (MeDIP-seq) to reveal the difference in methylation between breast cancer drug-resistant cells and sensitive cells. A total of 55076 differentially methylated genes (DMGs) were detected, including 21061 hypermethylated DMGs and 34015 hypomethylated DMGs. Moreover, Gene Ontology (GO) analysis and KEGG pathway analysis reveal the function and pathway of screening genes. These results indicate that DNA methylation may be involved in regulating the occurrence and development of breast cancer.

## Introduction

Cancer is one of the main diseases threatening human health. Among the different cancer parts from human tissues and organs, breast cancer is the most common cancer among women and one of the leading causes of cancer-related death of worldwide [1]. It is considered to be a multifactorial disease with extensive genetic mutations and chromosomal abnormalities. What’s more, the development of it also depends on epigenetic factors [2]. The principal strategies for clinical treatment of breast cancer include surgery combined with radiotherapy and chemotherapy. Taxol is one of the most widely used chemotherapeutic agents for the treatment of breast cancer [3]. It interferes with the depolymerization of microtubules in tumor cells, causing cell cycle arrest, preventing cancer cells from replicating, and ultimately leading to cell death [4]. However, breast cancer patients develop resistance to Taxol, which led to treatment failure. To improve the prognosis of patients, it is urgent to find the molecular mechanism leading to drug-resistance.

Epigenetics involves three molecular events: DNA methylation, histone modification, and chromatin remodeling. Among them, DNA methylation is one of the most widely studied, stable, and important heritable epigenetic modifications [5]. It occurs preferentially at the 5 'position of cytosine in CpG dinucleotides, which is mainly present in CpG islands (CGI) [6, 7]. It is also engaged in a variety of important biological processes, such as gene expression regulation, genomic imprinting, X chromosome inactivation, suppression of repetitive elements and carcinogenesis [8–10]. Moreover, abnormal DNA methylation is not only a feature of early carcinogenesis but also a feature of resistance to chemotherapeutic drugs, which make it a promising candidate biomarker [11, 12]. However, a lot of researches have focused on the genetic basis of breast cancer, and little know about the effects of epigenetic mechanisms on the development and prognosis of breast cancer [13–15]. Therefore, we performed DNA methylation profiling analysis of breast cancer drug-resistant cells MCF-7 / Taxol and sensitive cells MCF-7 to understand DNA methylation changes that occurred in the disease phenotype.

In this study, we took the first time to depict the whole-gene DNA methylation profile using methylated DNA immunoprecipitation-sequencing (MeDIP-seq) in breast cancer drug-resistant cells MCF-7 / Taxol and sensitive cells MCF-7 to gain a better understanding of the contribution of DNA methylation to the treatment of breast cancer. Our results showed that the distribution of DNA methylation peaks in the breast cancer cell genome, and identify hypermethylated and hypomethylated genes in breast cancer cells. In addition, our results also determined that the potential role of DNA methylation in gene expression in drug-resistant cells. This discovery will raise our level of understanding of breast cancer methylation group.

## Materials and methods

### Cell culture

The human breast cancer cells (MCF-7) were purchased from the American Type Culture Collection (ATCC, USA). Low concentration gradient induction method was used to establish breast cancer Taxol-resistant cells MCF-7 / Taxol. MCF-7 cells were cultured in a complete RPMI 1640 (Solarbio, China) medium containing 10% fetal bovine serum (BI, USA). MCF-7 / Taxol cells were stably cultured in complete RPMI 1640 medium containing 85.50 nmol• L^-1^ Taxols (Xi 'an Haoxuan bio-tech Co., Ltd., China). All cells were cultured aseptically in a humidified atmosphere of 5%CO_2_ at 37° C.

### MeDIP-seq library construction and sequencing

In this study, methylated DNA fragments were enriched by immunoprecipitation with a monoclonal antibody against 5-methylcytidine (5mC) using methylated DNA immunoprecipitation-sequencing (MeDIP-seq) technology [16, 17]. According to the manufacturer's instructions, DNA was extracted from the cells and broken into fragments of 200–800 bp. The production of small fragments is the key to ensuring effective immunoprecipitation and reasonable resolution, which is necessary for further testing [18]. Following the manufacturer’s instructions, 1 μg of fragmented sample was ligated to Illumina’s genomic adapters with Genomic DNA Sample Kit (#FC-102-1002, Illumina). Ligated DNA fragments were further immunoprecipitated by anti-5-Methylcytosine antibody (Diagenode). The enriched DNA was amplified by PCR and purified by AMPure XP beads. Quality control was performed to evaluate the quality of the MeDIP experiment.

The DNA fragments in the well-mixed library were denatured with 0.1M NaOH to generate single-stranded DNA molecules, loaded onto the channels of the flow cell at 8 pM concentrations, and amplified in situ. Sequencing was carried out using the Illumina NovaSeq 6000 by running 150 cycles according to the manufacturer's instructions.

### Bioinformatics

The raw sequencing data were processed to filter out low-quality reads. The trimmed reads were aligned to reference genome with Hisat2 software. Based on alignment statistical analysis (mapping ratio), we determine whether the results can be used for subsequent data analysis. If possible, aligned reads were used for peak calling, MeDIP enriched regions (peaks) with statistically significant were identified for each sample, using a q-value threshold of 10–5 by MACS2 [19]. We also analyzed the peak distribution of different genomic components in each group of cells, including genebody, intergenic, and promoter regions. Then we analyzed DMRs between breast cancer drug-resistant cells and sensitive cells, and then identified DMGs using DMR data. Functional enrichment analysis of GO and KEGG pathways was performed on the selected genes (p <0.05). In addition, to further understand the interaction between the critical pathways, the STRING database was used to generate a network structure of genes involved in KEGG pathways. Afterwards, a visual PPI network was constructed by Cytoscape software (version 3.7.2, http://www.cytoscape.org/) and the candidate hub genes were determined. Then we used online tools The Human Protein Atlas (HPA) (https://www.proteinatlas.org/) and UALCAN (http://ualcan.path.uab.edu/index.html) to verify the expression and prognosis of the candidate central genes.

### MeDIP-qPCR

We randomly selected three genes (POLD3; NR3C1; OTUB2) in the promoter regions for MeDIP-qPCR. According to the manufacturer's instructions, MeDIP technology was used to enrich methylated DNA fragments, and the enrichment of the 5mC-containing DNA sequences was quantified by real-time PCR. Calculate the frequency of DNA methylation between immunoprecipitated DNA and input DNA. Table 1 list of the primers used for MeDIP-qPCR.

**Table 1:** Polymerase Chain Reaction (PCR) primers.

Name	Forward primer	Reverse primer
POLD3	5'-CATTGGTATTTCTTGGCTTGT-3'	5'-GTCATATTGGAGTAGGGTGGA-3'
NR3C1	5'-GCTGGCGACACTGTACCCTA-3'	5'-CCCCTGCTCTGACATCTTGAA-3'
OTUB2	5'-GGGTCGCCTCCTCTTTGTTA-3'	5'-CCCCGTGCAACCCCTAGAT-3'

### Statistical analysis

SPSS 23.0 software was used for statistical analysis. All data were obtained from at least three independent experiments and expressed as mean ± SD. Comparisons between two groups were made with Student's t-test. For all data, P <0.05 was considered statistically significant.

## Results

### Genome-wide MeDIP-seq analysis of breast cancer cells

In order to analyze the DNA methylation pattern of breast cancer cells, we used breast cancer drug-resistant cells MCF-7 / Taxol (n = 3) and sensitive cells MCF-7 (n = 3) for MeDIP-seq analysis. According to the manufacturer's instructions, extract DNA from the cells and interrupt it with ultrasound. We observed that the size of the DNA after disruption was between 200–800 bp (S1 Fig). Then the library was constructed with the broken DNA and sequencing was carried out using the Illumina NovaSeq 6000 according to the manufacturer’s instructions. To assess the sequencing quality, the quality score plot of each sample was plotted (S1 Table). Generally, the percentage of the number of bases with Q30 should be greater than 80%.

After removing the adapter sequences, contamination, and low-quality reads from the original MeDIP-seq data, an average of 10.6 Gb clean reads per sample was obtained. Then we mapped the all reads to the reference genome. In MCF-7 / Taxol cells, 85.45, 82.67 and 84.05% reads were aligned to the reference genome. In MCF-7 cells, 83.25, 85.37 and 84.18% reads were mapped to the reference genome. The mapping rate ranged from 82.67% to 85.45% (S2 Table). S2 Fig displayed the distribution of the MeDIP-seq signal in each chromosome in breast cancer drug-resistant cells MCF-7 / Taxol and sensitive cells MCF-7. Uniquely mapped reads were detected on all chromosomes (chromosome 1–22 and X chromosome).

### Different DNA methylation patterns between two types of breast cancer cells

**Fig 1:**
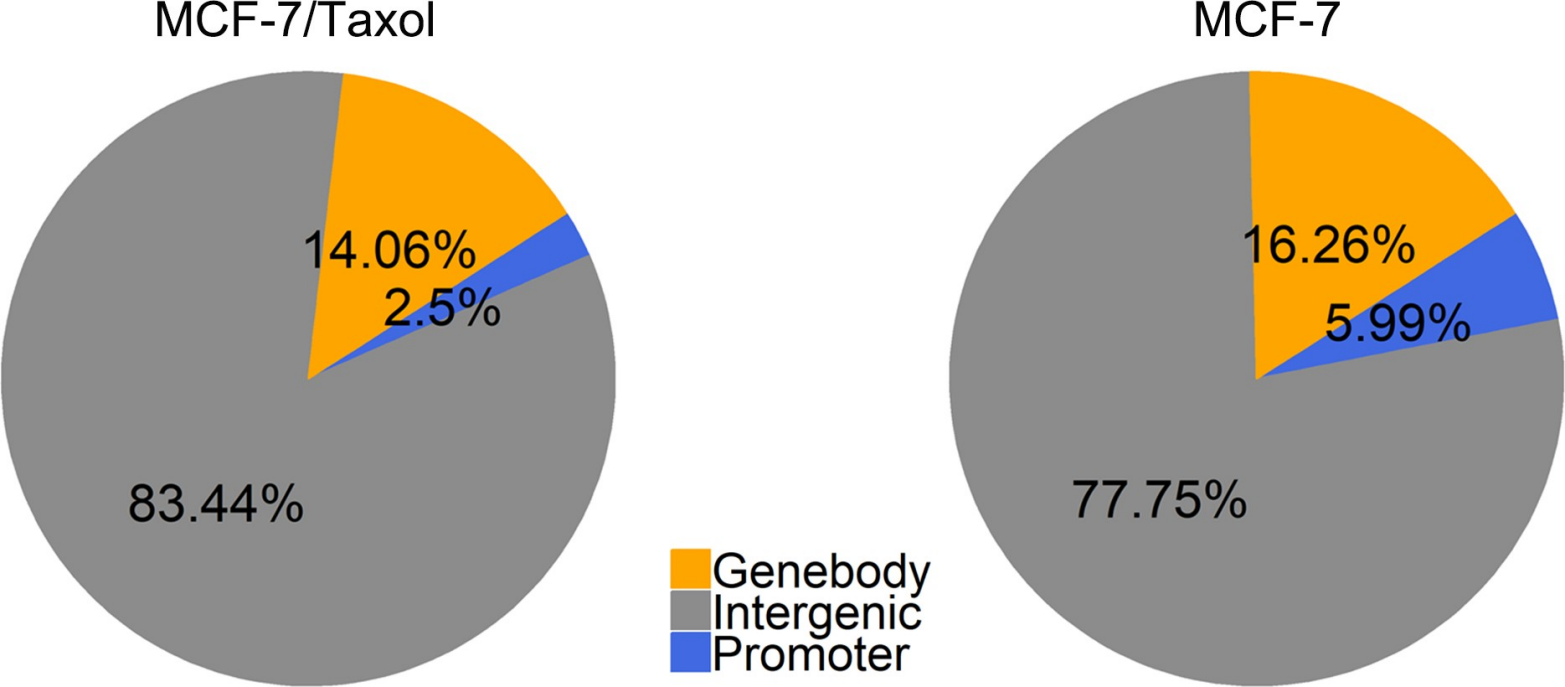
Different DNA methylation patterns in breast cancer cells. Genomic DNA was isolated from cultured breast cancer drug-resistant cells MCF-7 / Taxol and sensitive cells MCF-7 for MeDIP-seq analysis. As shown, methylation enrichment peaks were distributed in the genebody, intergenic, and promoter regions.

The mapped reads were used for the detection of statistically significant methylated regions. MeDIP enriched peaks with statistically significant were identified for each sample, using a q-value threshold of 10^−5^ by MACS2 software. We analyzed the distribution of methylation enriched peaks in different regions of the genome and found that they mainly exist in intergenic region (Fig 1). Compared with MCF-7 cells, we observed that the overall methylation level and regional methylation level of MCF-7 /Taxol cells were lower (S3 Table). These results indicated that DNA methylation patterns were different between the two groups of cells. Moreover, many studies have shown that the methylation patterns of breast cancer resistant cells and sensitive cells are different [20–22]. This information also proves the reliability of our research results.

### Differentially Methylated Regions (DMRs) of breast cancer drug-resistant cells and sensitive cells

**Fig 2:**
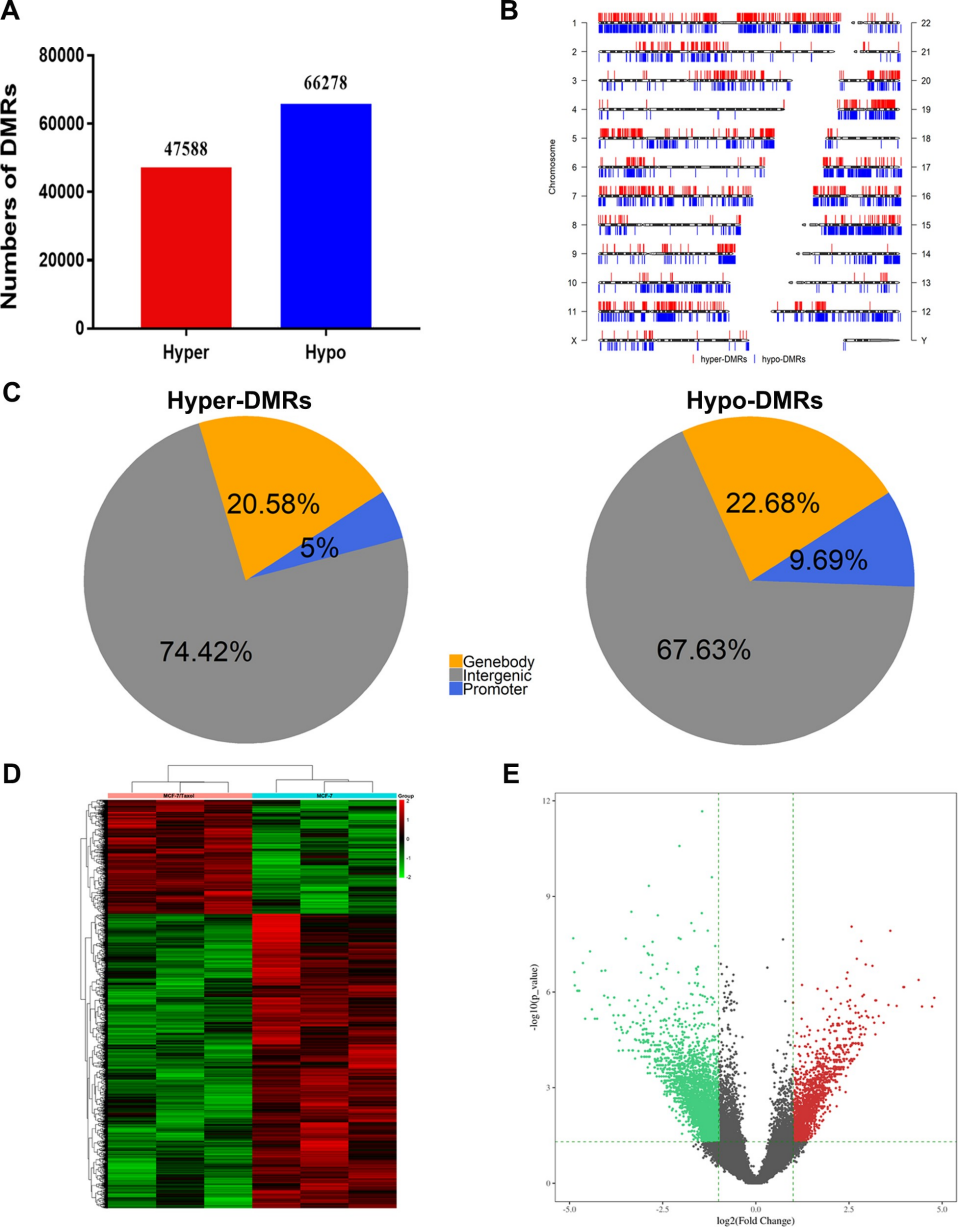
Difference methylated region of breast cancer drug-resistant cells and sensitive cells. A. Total hypermethylated and hypomethylated DMRs in MCF-7/Taxol and MCF-7 cells. B. Physical positions of DMRs in chromosomes. C. Distributions of DMRs in different elements of the genome. D. Heat map of the differential methylated region. Each region corresponds to each row. E. A volcanic map of the differential methylated region. Red points represent upregulated reads, green points represent downregulated reads, and black points are not statistically significant.

We analyzed differentially methylated regions (DMR) in breast cancer drug-resistant cells MCF-7 / Taxol and sensitive cells MCF-7. A total of 113,866 DMR (P <0.05, fold change≥2) were identified using diffReps software (S4 Table). Among them, 47588 (41.8%) were hypermethylated and 66278 (58.2%) were hypomethylated (Fig 2A). Next, we describe the distribution of DMRs in all chromosomes. As showed in Fig 2B, hypermethylation and hypomethylation DMR signals were mapped to the entire genome. This was consistent with what we observed in the overall DNA methylation pattern. To further compare the DNA methylation profiles of the two groups of breast cancer cells, we examined the genomic distribution of hypermethylated and hypomethylated DMRs in different chromosomal regions. It can be seen that most DMRs were located in the intergenic region (Fig 2C). These DMRs showed different patterns in breast cancer drug-resistant cells MCF-7 / Taxol and sensitive cells MCF-7.

Promoter methylation is known to be important in controlling gene expression [23, 24]. Moreover, it is recognized that promoter methylation is involved in the development of cancer [25]. Next, we focus on the analysis of DMR in the promoter region. As shown in Fig 2D, there were 8804 DMRs between MCF-7 / Taxol cells and MCF-7 cells, including 2381 hypermethylated regions and 6423 hypomethylated regions. Lower levels of methylation were observed in MCF-7 / Taxol cells. In a volcano plot (Fig 2E), the distribution of hypermethylated and hypomethylated DMRs was in the quadrant with fold change≥ 2 and P <0.05, and we could see that the larger DMRs was concentrated in the hypomethylated quadrant. Therefore, drug-resistant cells tend to be hypomethylated.

### Differentially Methylated Genes (DMGs) of breast cancer drug-resistant cells and sensitive cells

**Fig 3:**
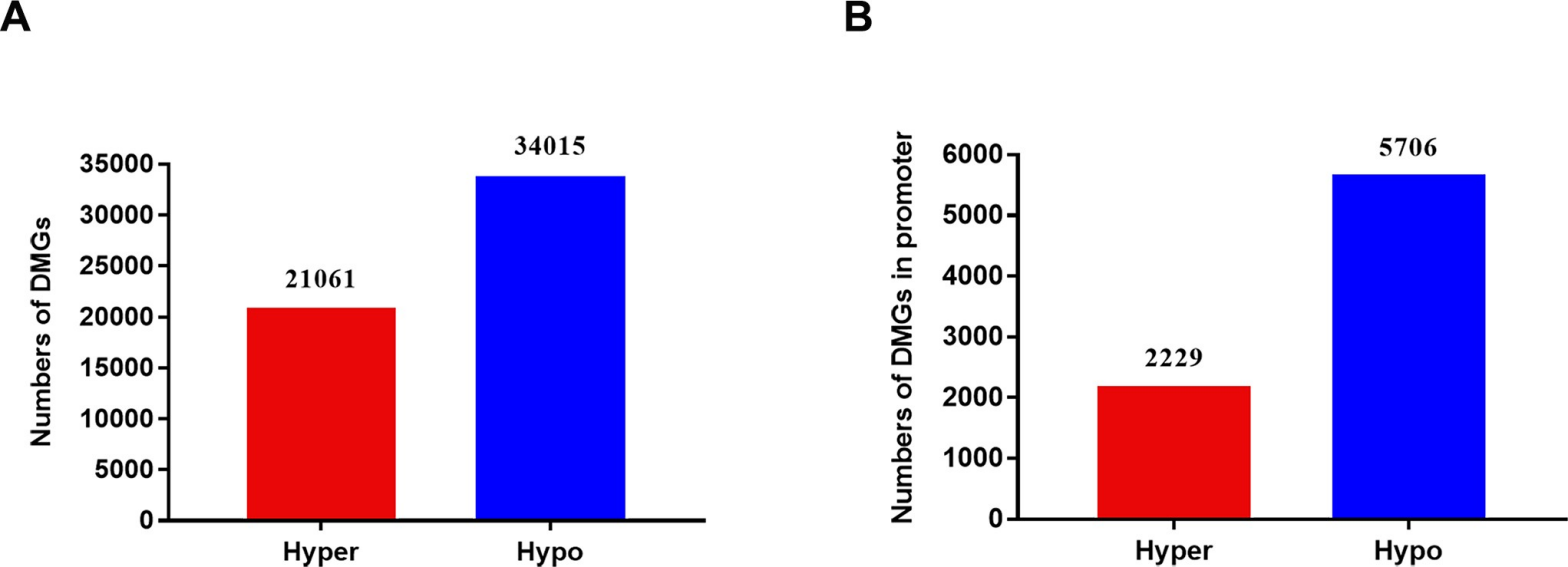
Distributions of differentially methylation genes in MCF-7/Taxol cells and MCF-7 cells. A. Numbers of Differentially Methylated Genes in the whole genome. B. Numbers of Differentially Methylated Genes in the promoter region.

Using DMR data to identify DMG, we identified genes containing DMR in two groups. We detected 55076 DMGs in breast cancer drug-resistant cells MCF-7 / Taxol and sensitive cells MCF-7, including 21061 hypermethylated DMGs and 34015 hypomethylated DMGs (Fig 3A). After filtering out DMG with DMR only located in the promoter region, we identified 2229 hypermethylated DMG and 5706 hypomethylated DMG in MCF-7 / Taxol cells compared with MCF-7 cells (Fig 3B). We can see that the distribution of DMGs in the promoter region was similar to that of genome-wide DMGs. That is, drug-resistant cells have a tendency of hypomethylation.

### Validation of MeDIP-Seq data through MeDIP-qPCR

In order to verify the reliability of the MeDIP-Seq results, DMGs (POLD3; NR3C1; OTUB2) in the promoter region were randomly selected for MeDIP-qPCR. The results showed that the 5mC levels of CGIs in the POLD3 and NR3C1 promoters were significantly increased in MCF-7/Taxol cells compared with MCF-7 cells. The 5mC levels of CGIs in the OTUB2 promoter were significantly decreased in MCF-7/Taxol cells compared with MCF-7 cells. These results were consistent with the MeDIP-Seq results (S3 Fig).

### Gene Ontology (GO) enrichment analysis of DMGs

**Fig 4:**
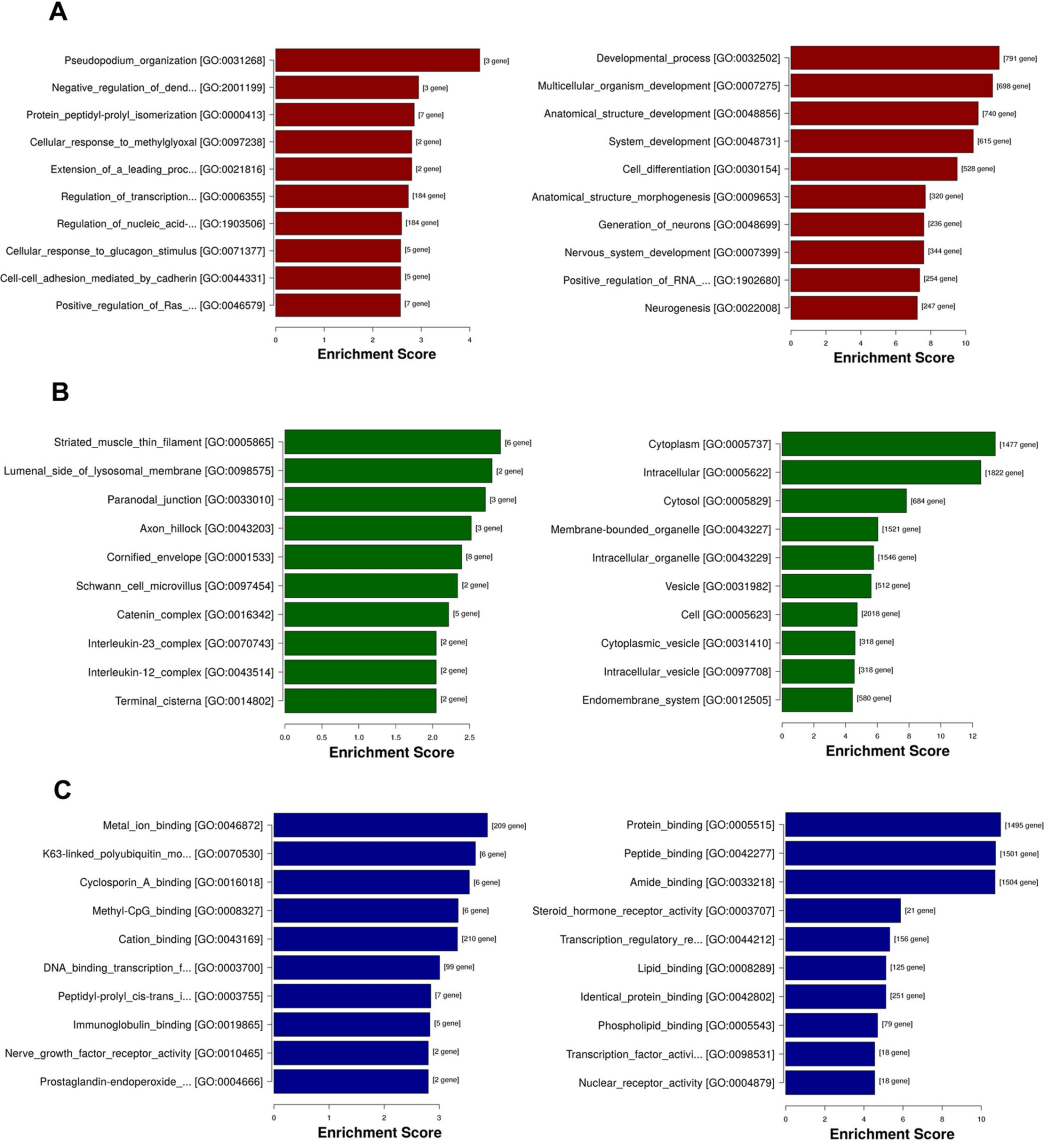
GO enrichment analysis of differentially methylation genes in the promoter region. A. biological process; B. cellular component; C. molecular function. The bar plot showed the top ten enrichment score values of the significant enrichment terms. The left panel showed the GO terms involved in hypermethylated DMGs. The right panel showed the GO terms involved in hypomethylated DMGs.

To determine the biological function of DMGs in the promoter region of the two groups of cells, GO analysis was performed. GO annotation data can be divided into three categories: biological processes, cellular components, and molecular functions. The biological processes involved 728 hypermethylated and 2111 hypomethylated DMGs, cellular components involved 769 hypermethylated and 2179 hypomethylated DMGs, and molecular functions involved 720 hypermethylated and 2099 hypomethylated DMGs. Details of GO enrichment analysis was listed in S5 Table. GO enrichment analysis was conducted to gain a deeper understanding of the biological processes, cellular components, and molecular functions that DMGs may be involved in. Fig 4 showed the top ten enrichment score values of the significant enrichment terms (P <0.05). These categories may regulate the sensitivity of cells to chemotherapy drugs through DNA methylation.

### Pathway and path network analysis of DMGs

**Fig 5:**
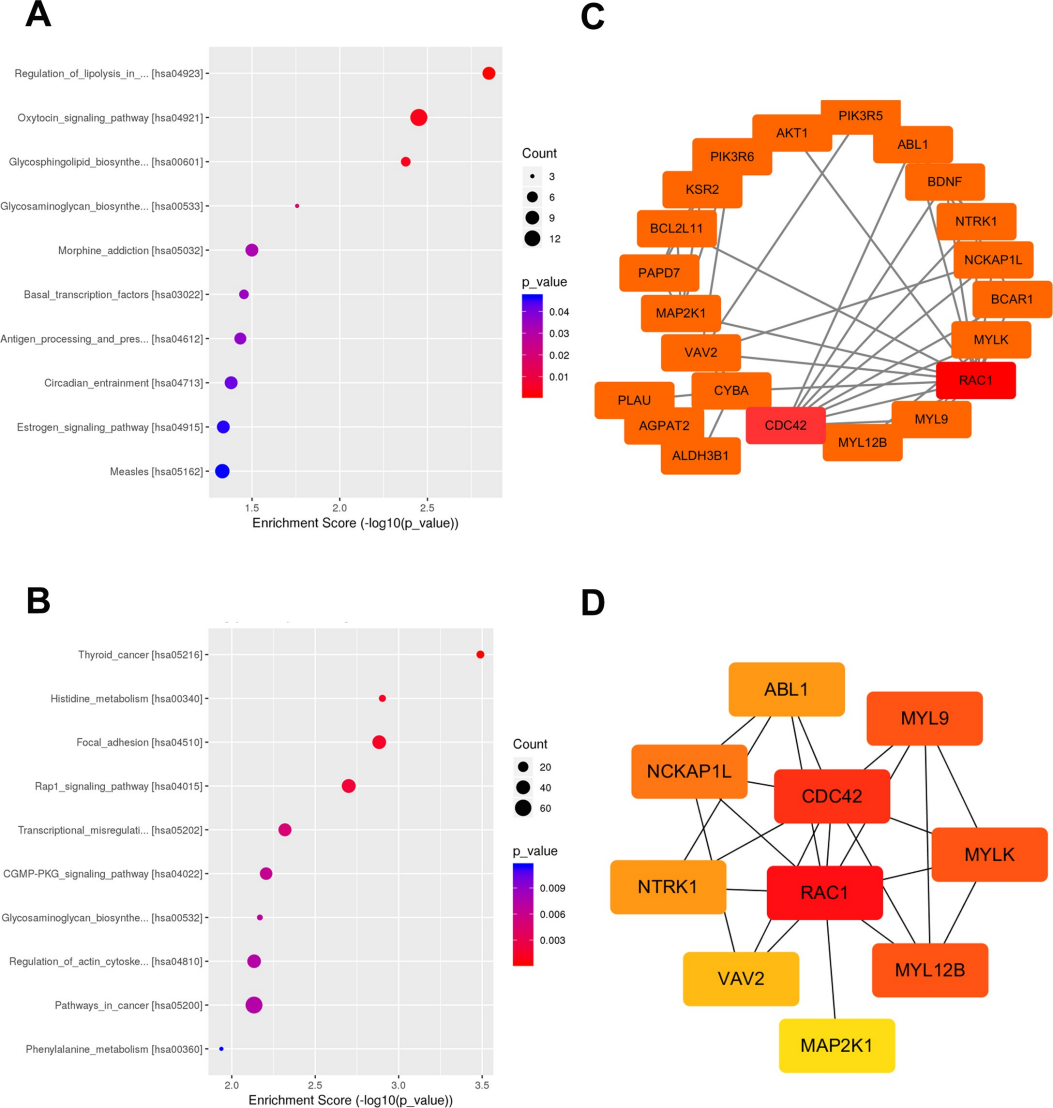
KEGG pathway and path network of differentially methylation genes. A. The dot plot showed the top ten enrichment score values of the significant enrichment pathway of hypermethylated DMGs. B. The dot plot showed the top ten enrichment score value of the significant enrichment pathway of hypomethylated DMGs. Dot size reflected the number of genes enriched in each signaling pathway. Dot color indicated P-value. C. The PPI network for the 22 genes involved in the functional module of a highest score. D. The top ten hub genes scoring in the PPI network.

**Fig 6:**
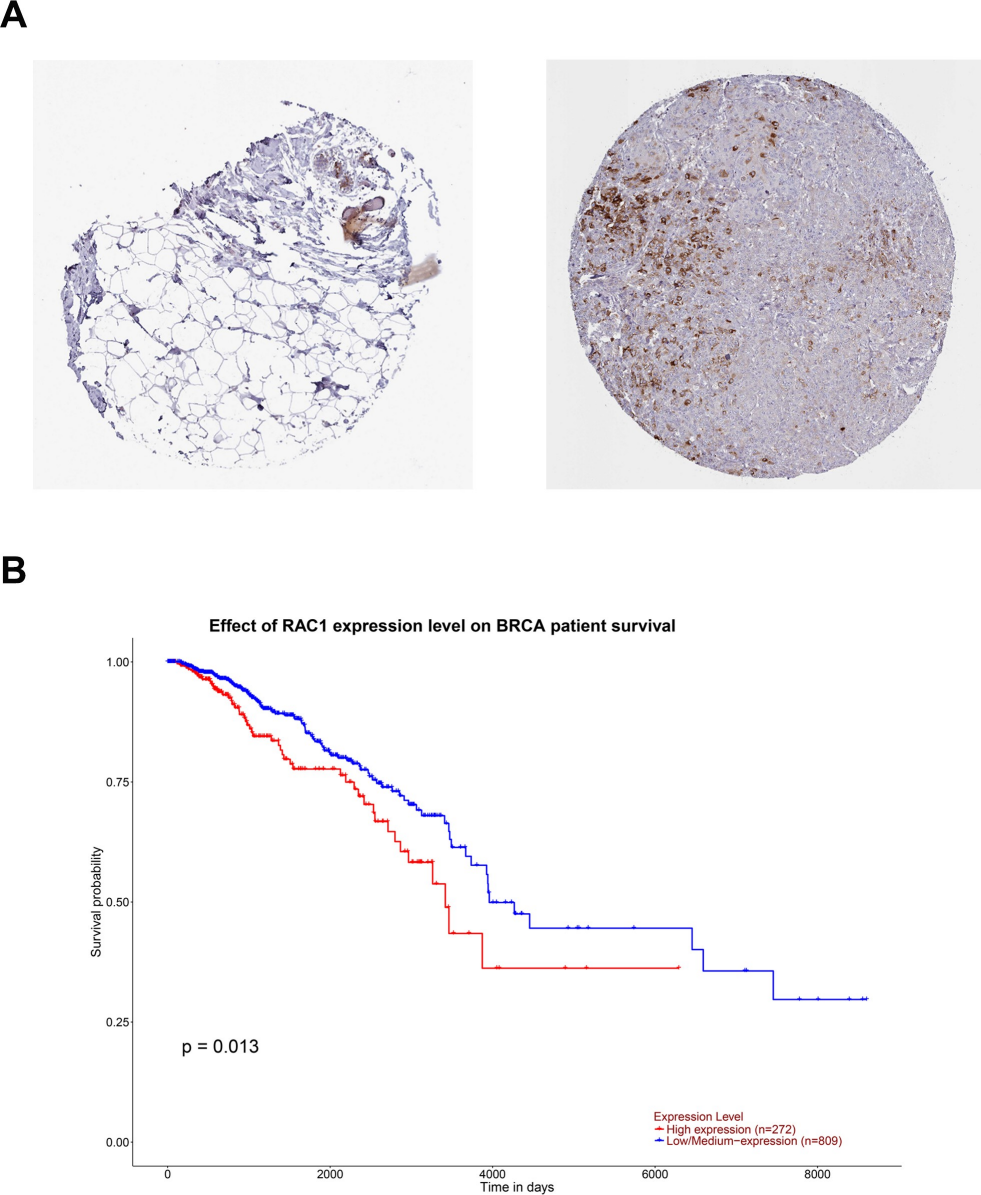
KEGG pathway and path network of differentially methylation genes. A. Immunohistochemistry map of the candidate central gene (RAC1) protein expression in normal and BC tissues. B. Survival analysis of RAC1 in BC. *P* < 0.05 was considered as statistically significant. The blue line represents the low expression of RAC1; red line represents the high expression of RAC1.

To determine the important pathways involved in DMGs, we performed the KEGG pathway to predict the putative function of DMGs in the promoter region. The output results showed that hypermethylated DMGs were significantly enriched in 11 pathways (P <0.05, S6 Table), the most important of which were “Regulation of lipolysis in adipocytes, Oxytocin signaling pathway, Glycosphingolipid biosynthesis-lacto and neolacto series, Glycosaminoglycan biosynthesis-keratan sulfate, and Morphine addiction” (Fig 5A). Hypomethylated DMGs were closely related to 23 pathways (P <0.05, S7 Table), the most important of which were “Thyroid cancer, Histidine metabolism, Focal adhesion, Rap1 signaling pathway and Transcriptional misregulation in cancer” (Fig 5B). To further understand the interaction between the critical pathways of DMGs, the STRING database was used to generate a network structure of genes involved in KEGG pathways (S4 Fig). Then the PPI network was visualized by Cytoscape software. Using the plugin "MCODE" to search for the functional module, we finally obtain a module composed of 22 genes with the highest score (Fig 5C). After that, the top ten hub genes scoring was then identified using the plugin "cytoHubba" (Fig 5D). Based on the online tool HPA, the immunohistochemical map of candidate central gene (RAC1) protein expression is shown in Fig 6A. We can see that the expression level of RAC1 is higher in breast cancer tissue. We also analyzed the prognostic value of the RAC1 using online tools UALCAN. As showed in the Fig 6B, the survival analysis shows that the prognosis of breast cancer patients with high expression of RAC1 is worse than that of patients with low expression (P<0.05). Moreover, RAC1, CDC42, MYL9, MYLK, ABL1, and other genes have been proved to be related to the progress of human breast cancer [26–31]. These data clearly provide useful information for the resistance development of breast cancer.

## Discussion

Drug-resistant and sensitive cells have a different death pattern in breast cancer. When chemotherapy drugs act on MCF-7 cells, they undergo pyroptosis, which is manifested by the formation of membrane pores, the release of cellular contents, and the occurrence of inflammatory reactions [32]. However, chemotherapeutic drugs apply to MCF-7 / Taxol cells, they develop apoptosis, showing resistance to chemotherapeutic drugs [33, 34]. Obviously, intrinsic and acquired chemotherapy resistance is the main reason for the prognosis of breast cancer patients. The mechanism of cells acquire this resistance is still largely unclear, but many studies have shown that DNA methylation patterns are different in breast cancer drug-resistant cells and sensitive cells [35–37]. In order to study the changes of DNA methylation in cells, in this study, we used a low concentration gradient induction method to induce breast cancer sensitive cells MCF-7 into Taxol-resistant cells MCF-7 / Taxol (S5 Fig), and analyze the genome-wide DNA methylation pattern of breast cancer drug-resistant cells and sensitive cells by MeDIP-seq.

Several methods have been designed for the analysis of genome-wide DNA methylation profiles, including whole-genome bisulfite sequencing (WGBS), methylated DNA immunoprecipitation-sequencing (MeDIP-seq), and high-performance liquid chromatography (HPLG) [38–40]. Although bisulfite sequencing has been widely used for DNA methylation analysis, it is less cost-effective. The combination of MeDIP and high-throughput sequencing enables high-resolution analysis of methylomes, which currently is the best available method for genome-wide DNA methylation analysis [41, 42]. It firstly uses an anti-5-methylcytosine antibody to specifically recognize methylated cytosine and then enriches the methylated DNA fragments. In this study, we used MeDIP-seq to describe the genome-wide DNA methylation pattern of breast cancer cells.

For more than a decade, changes in cancer epigenetics have been the preface to epigenetic research [18]. Cancer epigenetics refers to the study of various epigenetic modifications to cancer cells [43]. Among the patterns of epigenetic modifications of cancer, abnormal DNA methylation patterns have been proven to be the most common and important pathogenesis of various cancers. Moreover, changes in DNA methylation profiles are a hallmark of almost all human cancers, including breast cancer. In this study, we used MeDIP-seq analysis technology to report for the first time the genome-wide DNA methylation profiles of breast cancer drug-resistant cells MCF-7 / Taxol and sensitive cells MCF-7. We concluded that the methylation patterns were different between the two groups of cells. 113,866 DMRs were identified, including 47,588 hypermethylated DMRs and 66,278 hypomethylated DMRs. Based on the DMRs data, we identified a total of 21,061 DMGs with hypermethylated and 34,015 DMGs with hypomethylated. Since the typical DMGs in the promoter is more suitable for verification by MeDIP-qPCR, we randomly selected genes (POLD3; NR3C1; OTUB2) in this region to check. These results were in agreement with the MeDIP-Seq results (S3 Fig).

Our results indicate that DNA methylation patterns are diverse in different treatment stages of the same cell. The distribution of reads is highest in the intergenic region, followed by the genebody and promoter regions. To investigate the potential biological function of DMGs, genes containing DMRs were selected for GO and KEGG pathway analysis. After filtering out DMGs using DMRs located only in the promoter region, we depicted biological processes involved in DMGs (Fig 4). Through analyzing the pathways of DMGs, we obtained several important pathways, including “Regulation of lipolysis in adipocytes, Oxytocin signaling pathway, Glycosphingolipid biosynthesis-lacto and neolacto series, Glycosaminoglycan biosynthesis-keratan sulfate, Morphine addiction, Thyroid cancer, Histidine metabolism, Focal adhesion, Rap1 signaling pathway, and Transcriptional misregulation in cancer”. More importantly, we conducted the network structure of DMGs involved in these pathways and screened out some molecules that play a major role in the development of breast cancer (Fig 5). We can know that the pathway analysis of DMGs provide a direction for understanding the resistance development of breast cancer, which not only helps us understand the role of DNA methylation in breast cancer but also helps us determine treatment targets.

In summary, we have completed the genome-wide map of breast cancer drug-resistant cells MCF-7 / Taxol and sensitive cells MCF-7, which can lay the foundation for further research on epigenetic regulation of breast cancer.

## Conclusion

To sum up, this study provides genome-wide DNA methylation analysis of breast cancer drug-resistant cells and sensitive cells, and identifies some DMGs that may be potential regulators from sensitivity to drug-resistance development in breast cancer, providing strong evidence for the in-depth study of resistance development and valuable information for future epigenetic research.

## Supporting information

S1 Fig The fragment size distribution of DNA by agarose gel electrophoresis. Lane 1: Total DNA of sample MCF-7/Taxol-1, Lane 2: Total DNA of sample MCF-7/Taxol-2, Lane 3: Total DNA of sample MCF-7/Taxol-3, Lane 4: Total DNA of sample MCF-7-1, Lane 5: Total DNA of sample MCF-7-2, Lane 6: Total DNA of sample MCF-7-2.
(TIF)

S2 Fig Chromosome distribution of reads in MCF-7/Taxol cells and MCF-7 cells. Distribution of reads in chromosomes 1 to 22 and chromosome X of the human genome was shown in red for each sample. MeDIP-seq reads were plotted in 10 kb windows along the chromosome. (A): MCF-7/Taxol cells; (B): MCF-7 cells.
(TIF)

S3 Fig The 5-methylcytosine (5mC) levels of CGIs in POLD3, NR3C1 and OTUB2 promoters in breast cancer cells. The isolated genomic DNA was subjected to MeDIP analysis. After immunoprecipitation with an anti-5mC antibody, the enrichment of the 5mC-containing DNA sequences was quantification by real-time PCR. Calculate the relative amounts of 5mC-containing DNA sequences compared with the input in each group (n = 3 / group). Statistical analysis is performed using GraphPad Prism 7 software. The student's t-test is used to measure MCF-7 / Taxol cells and MCF-7 cells from three independent replicates experimenting. Asterisks indicate a significant difference compared with MCF-7 cells (P <0.05). *: P <0.05, **: P <0.01, ***: P <0.001.
(TIF)

S4 Fig The network structure of DMGs engaged in KEGG pathways. Network nodes represented proteins generated by gene expression, and lines represented interactions between paths. The more lines around a node, the more paths connected to it, and the more prominent role it plays in the network.
(TIF)

S5 Fig MCF-7/Taxol cells are successfully induced into a highly resistant model. A. Cell proliferation was determined by CCK-8 assay after treatment with Taxol for 24, 48 and 72h. If the Resistance Index of cells are greater than 15, it indicates that they are highly resistant to drugs (Resistance Index (RI) = IC50(MCF-7/Taxol) /IC50(MCF-7)). From the IC50 of these two cells at different time- points (24, 48 and 72h), it can be calculated that the Resistance Index (RI) of MCF-7 /Taxol cells was greater than 15. B. The expression levels of ABCB1 mRNA were determined by qRT-PCR in MCF-7 and MCF-7/Taxol cells. GAPDH was used as an internal control. C and D. The expression levels of PGP were determined by Western blotting in MCF-7 and MCF-7/Taxol cells. It can be seen from figures that the expression levels of multidrug resistance gene in MCF-7 / Taxol cells is higher than that in the control group. Data are expressed as the mean ± SD of three independent experiments. *P<0.05, **P<0.01, ***P<0.001 *vs* control group.
(TIF)

S1 Table Information of sequencing quality. Quality score Q is logarithmically related to the base calling error probability (P): Q = 10 log10 (P). Q30 means the incorrect base calling probability to be 0.001 or 99.9% base calling accuracy.
(DOCX)

S2 Table Mapping results of MeDIP-seq. (DOCX)

S3 Table Numbers of methylation enrichment peaks in different gene components. (DOCX)

S4 Table Numbers of differentially methylated regions in different gene components. (DOCX)

S5 Table (XLSX)

S6 Table (XLSX)

S7 Table (XLSX)
