# Supplementary figures and images for: Genome-wide DNA methylation analysis of breast cancer MCF-7 / Taxol cells with MeDIP-Seq
Source: PLoS One. 2020 Dec 11;15(12):e0241515. doi: 10.1371/journal.pone.0241515 (PMC7732127; doi:10.1371/journal.pone.0241515)

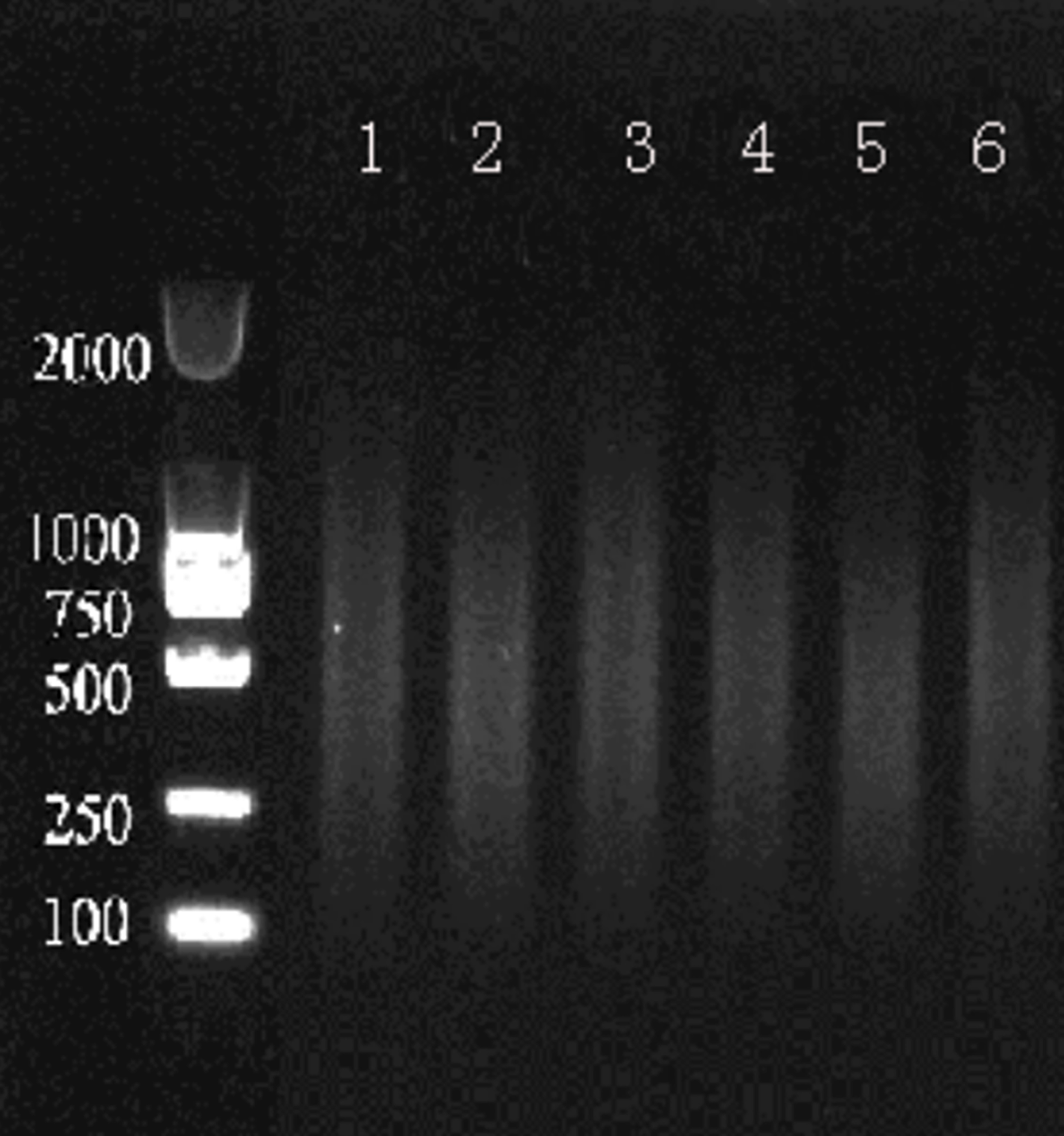

Supplement: S1 Fig — Lane 1: Total DNA of sample MCF-7/Taxol-1, Lane 2: Total DNA of sample MCF-7/Taxol-2, Lane 3: Total DNA of sample MCF-7/Taxol-3, Lane 4: Total DNA of sample MCF-7-1, Lane 5: Total DNA of sample MCF-7-2, Lane 6: Total DNA of sample MCF-7-2. (TIF) [file pone.0241515.s001.tif]

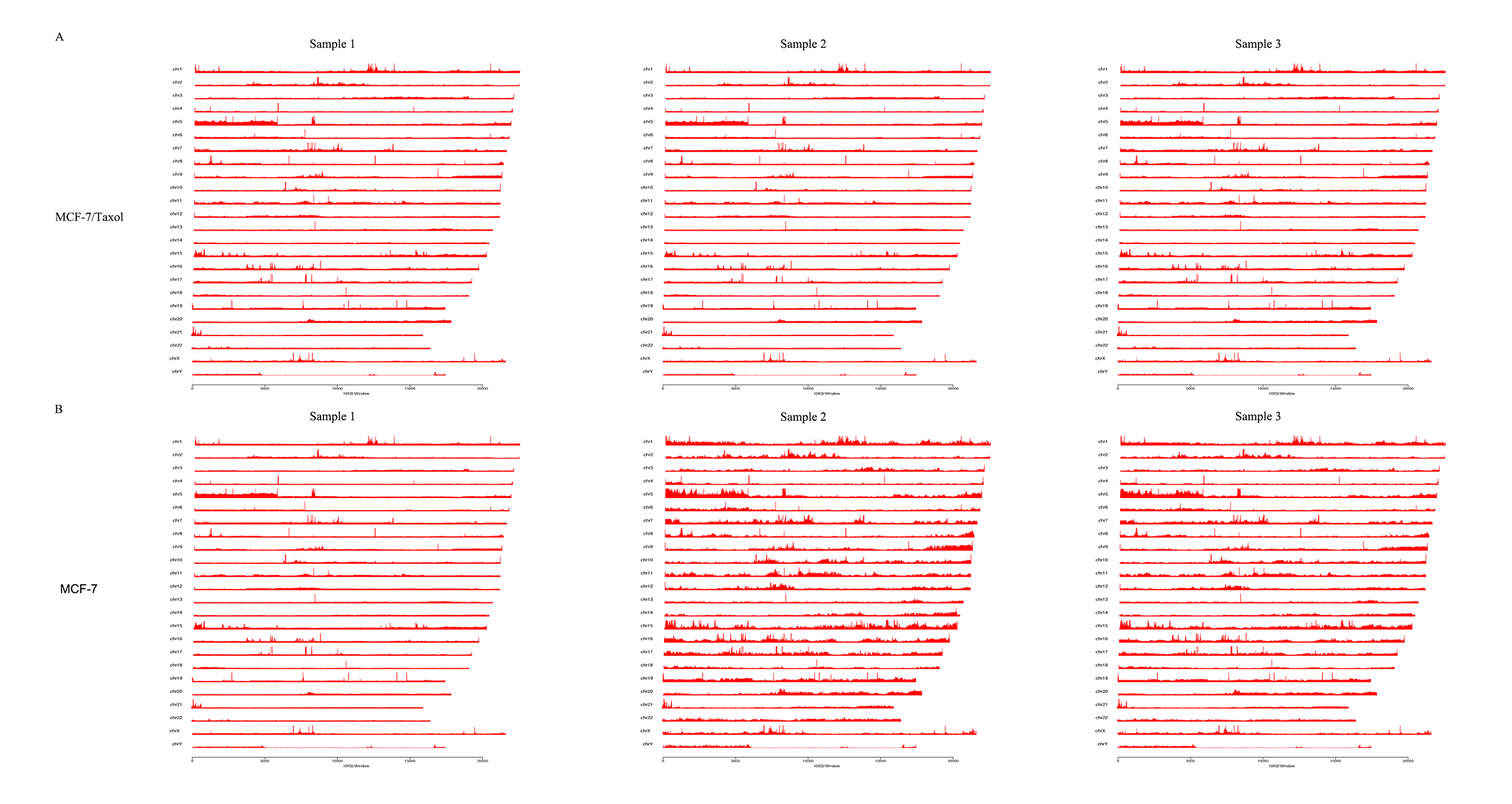

Supplement: S2 Fig — Distribution of reads in chromosomes 1 to 22 and chromosome X of the human genome was shown in red for each sample. MeDIP-seq reads were plotted in 10 kb windows along the chromosome. (A): MCF-7/Taxol cells; (B): MCF-7 cells. (TIF) [file pone.0241515.s002.tif]

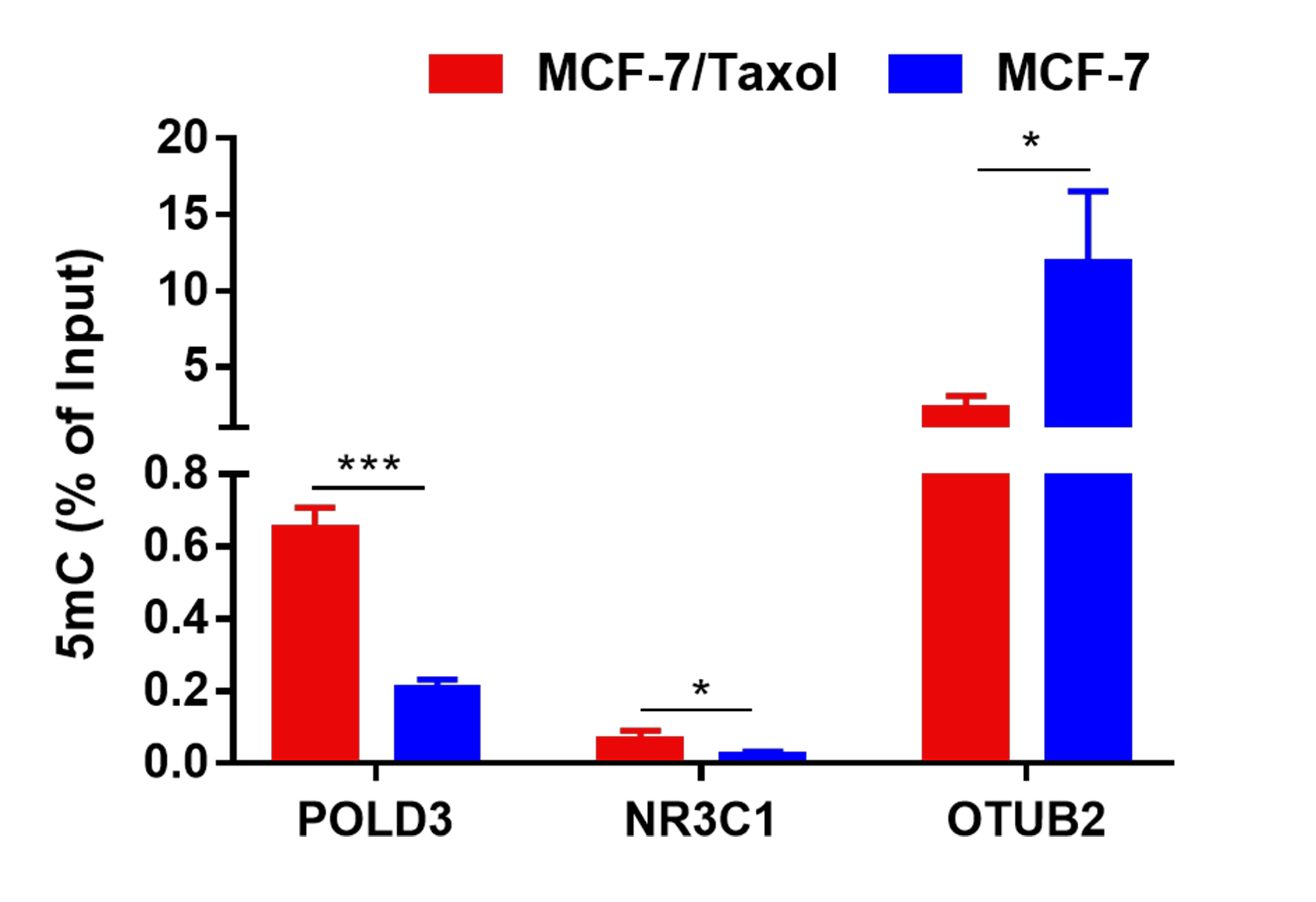

Supplement: S3 Fig — The isolated genomic DNA was subjected to MeDIP analysis. After immunoprecipitation with an anti-5mC antibody, the enrichment of the 5mC-containing DNA sequences was quantification by real-time PCR. Calculate the relative amounts of 5mC-containing DNA sequences compared with the input in each group (n = 3 / group). Statistical analysis is performed using GraphPad Prism 7 software. The student's t-test is used to measure MCF-7 / Taxol cells and MCF-7 cells from three independent replicates experimenting. Asterisks indicate a significant difference compared with MCF-7 cells (P <0.05). *: P <0.05, **: P <0.01, ***: P <0.001. (TIF) [file pone.0241515.s003.tif]

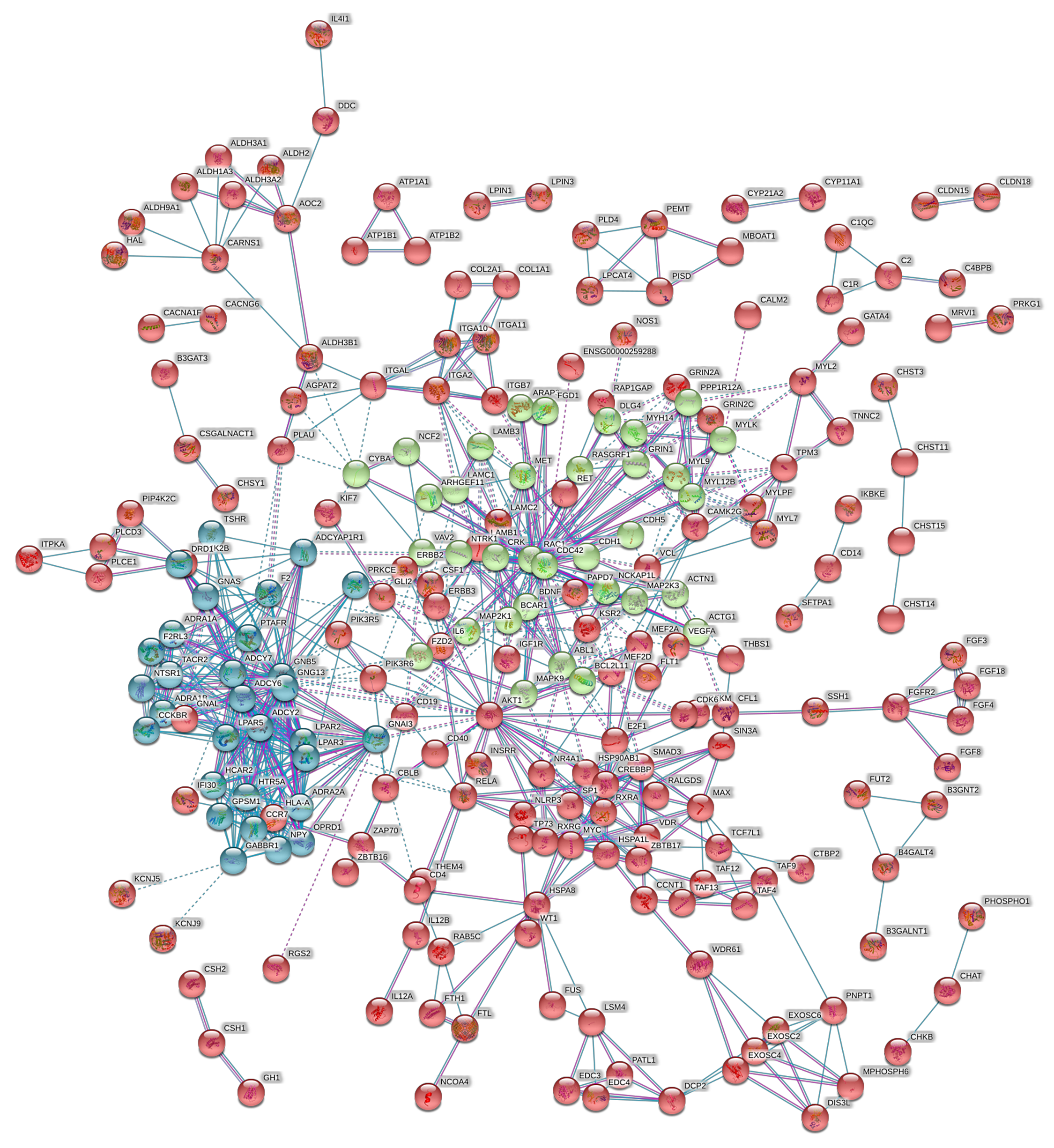

Supplement: S4 Fig — Network nodes represented proteins generated by gene expression, and lines represented interactions between paths. The more lines around a node, the more paths connected to it, and the more prominent role it plays in the network. (TIF) [file pone.0241515.s004.tif]

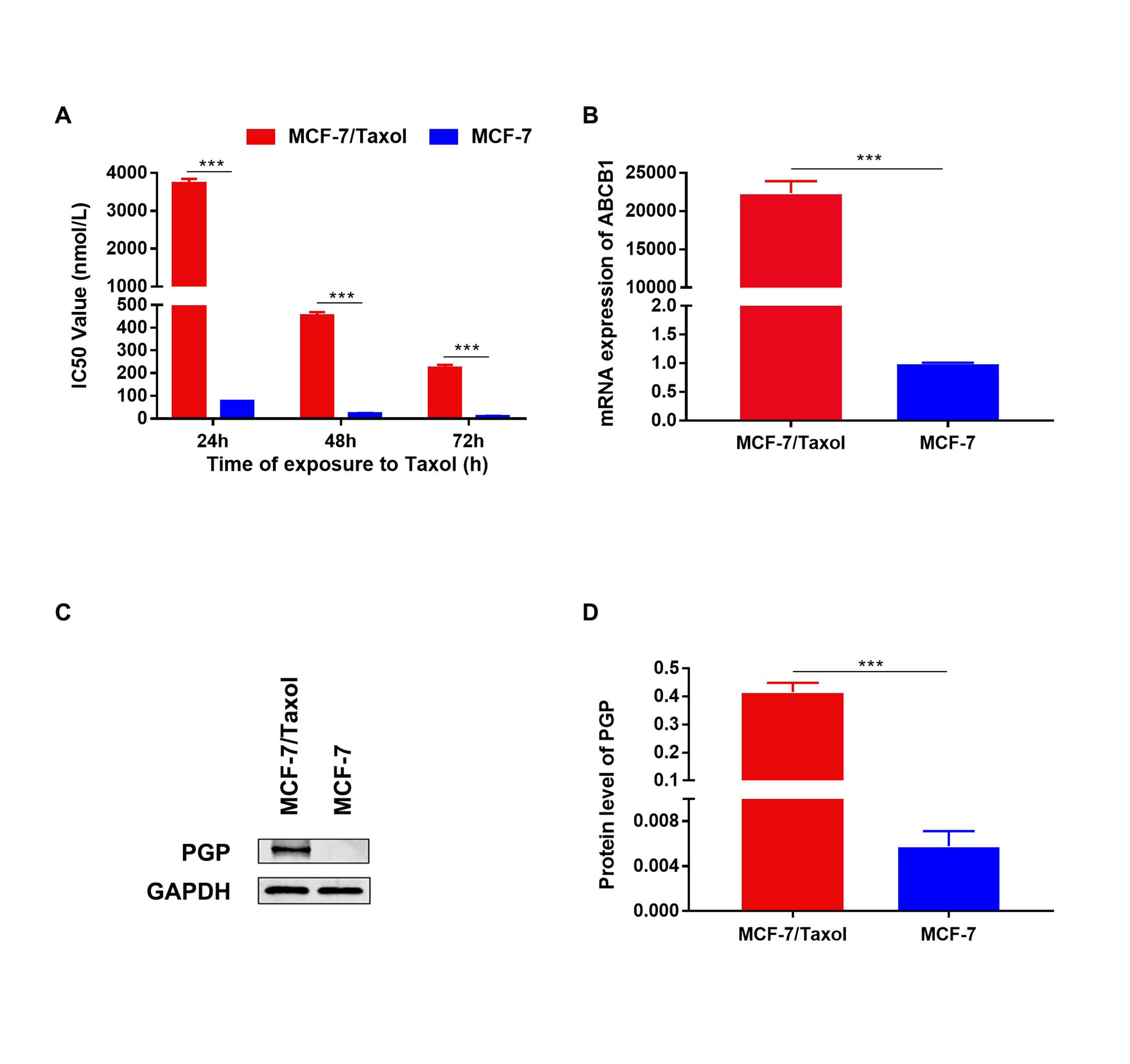

Supplement: S5 Fig — A. Cell proliferation was determined by CCK-8 assay after treatment with Taxol for 24, 48 and 72h. If the Resistance Index of cells are greater than 15, it indicates that they are highly resistant to drugs (Resistance Index (RI) = IC50(MCF-7/Taxol) /IC50(MCF-7)). From the IC50 of these two cells at different time- points (24, 48 and 72h), it can be calculated that the Resistance Index (RI) of MCF-7 /Taxol cells was greater than 15. B. The expression levels of ABCB1 mRNA were determined by qRT-PCR in MCF-7 and MCF-7/Taxol cells. GAPDH was used as an internal control. C and D. The expression levels of PGP were determined by Western blotting in MCF-7 and MCF-7/Taxol cells. It can be seen from figures that the expression levels of multidrug resistance gene in MCF-7 / Taxol cells is higher than that in the control group. Data are expressed as the mean ± SD of three independent experiments. *P<0.05, **P<0.01, ***P<0.001 vs control group. (TIF) [file pone.0241515.s005.tif]
