# Supplementary material for: Genome-wide DNA methylation analysis of breast cancer MCF-7 / Taxol cells with MeDIP-Seq
Source: PLoS One. 2020 Dec 11;15(12):e0241515. doi: 10.1371/journal.pone.0241515 (PMC7732127; doi:10.1371/journal.pone.0241515)
Supplement: S1 Table — Quality score Q is logarithmically related to the base calling error probability (P): Q = 10 log10 (P). Q30 means the incorrect base calling probability to be 0.001 or 99.9% base calling accuracy. (DOCX) [file pone.0241515.s006.docx]

**S1 Table: Information of Sequencing Quality.** Quality score Q is logarithmically

related to the base calling error probability (P): Q = 10 log10 (P). Q30 means the

incorrect base calling probability to be 0.001 or 99.9% base calling accuracy.

| Sample | | Reads Count | Bases Num | Bases Num (Q ≥30) | Q30 (%) |
| --- | --- | --- | --- | --- | --- |
| MCF-7/Taxol | 1 | 90095408 | 13514311200 | 12257070818 | 90.70 |
|  | 2 | 98250854 | 14737628100 | 13440049164 | 91.20 |
|  | 3 | 76056752 | 11408512800 | 10328218683 | 90.53 |
| MCF-7 | 1 | 74071948 | 11110792200 | 10101824533 | 90.92 |
|  | 2 | 60793720 | 9119058000 | 8190345069 | 89.82 |
|  | 3 | 77317978 | 11597696700 | 10369912706 | 89.41 |
