# Supplementary material for: Genome-wide DNA methylation analysis of breast cancer MCF-7 / Taxol cells with MeDIP-Seq
Source: PLoS One. 2020 Dec 11;15(12):e0241515. doi: 10.1371/journal.pone.0241515 (PMC7732127; doi:10.1371/journal.pone.0241515)
Supplement: S2 Table — (DOCX) [file pone.0241515.s007.docx]

**S2 Table: Mapping results of MeDIP-Seq.**

| Sample | | Raw Pairs | Mapped | Mapped (%) |
| --- | --- | --- | --- | --- |
| MCF-7/Taxol | 1 | 49117691 | 41973046 | 85.45 |
|  | 2 | 45046900 | 37239038 | 82.67 |
|  | 3 | 38026786 | 31960286 | 84.05 |
| MCF-7 | 1 | 37034940 | 30832332 | 83.25 |
|  | 2 | 30395825 | 25948663 | 85.37 |
|  | 3 | 38658841 | 32541163 | 84.18 |
