# Supplementary material for: Genome-wide DNA methylation analysis of breast cancer MCF-7 / Taxol cells with MeDIP-Seq
Source: PLoS One. 2020 Dec 11;15(12):e0241515. doi: 10.1371/journal.pone.0241515 (PMC7732127; doi:10.1371/journal.pone.0241515)
Supplement: S3 Table — (DOCX) [file pone.0241515.s008.docx]

**S3 Table: Numbers of Methylation Enrichment Peaks in different gene components.**

| gene region | Peak numbers | |
| --- | --- | --- |
|  | MCF-7/Taxol | MCF-7 |
| Genebody | 845.6666667 | 1537.333333 |
| Intergenic | 5081 | 7351.666667 |
| Promoter | 150.3333334 | 566.3333334 |
