# Supplementary material for: Genome-wide DNA methylation analysis of breast cancer MCF-7 / Taxol cells with MeDIP-Seq
Source: PLoS One. 2020 Dec 11;15(12):e0241515. doi: 10.1371/journal.pone.0241515 (PMC7732127; doi:10.1371/journal.pone.0241515)
Supplement: S4 Table — (DOCX) [file pone.0241515.s009.docx]

**S4 Table. Numbers of Differentially Methylated Regions in different gene components.**

| gene region | DMRs | |
| --- | --- | --- |
|  | Hyper | Hypo |
| Genebody | 9794 | 15031 |
| Intergenic | 35413 | 44824 |
| Promoter | 2381 | 6423 |
